# Supplementary figures and images for: Physiological changes and gene responses during Ganoderma lucidum growth with selenium supplementation
Source: PeerJ. 2022 Dec 20;10:e14488. doi: 10.7717/peerj.14488 (PMC9784338; doi:10.7717/peerj.14488)

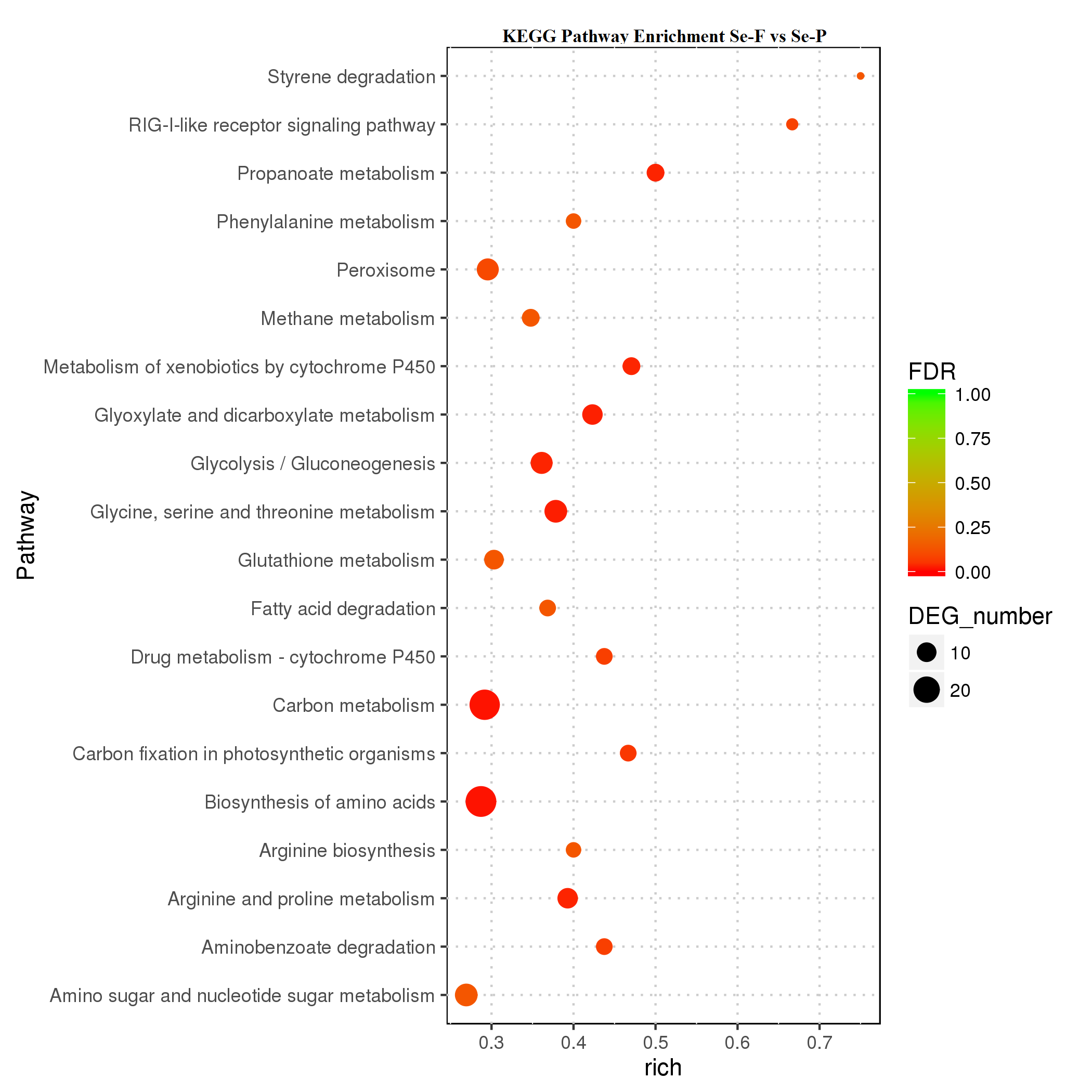

Supplement: Supplemental Information 1 [file peerj-10-14488-s001.zip › raw data/Se-P VS Se-F.png]

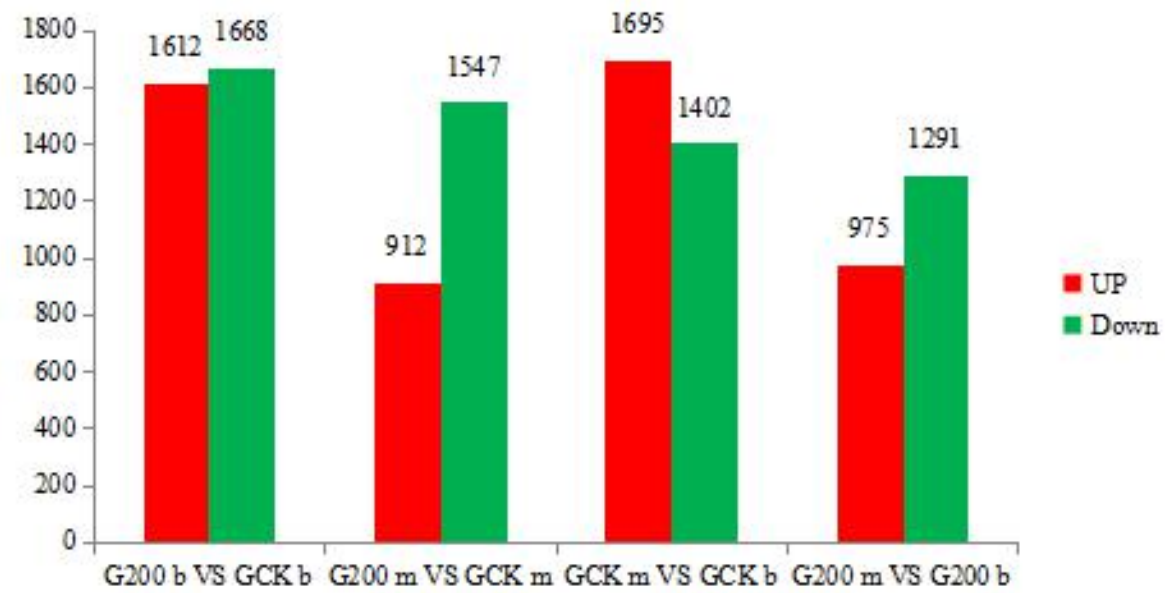

Fig. S1 Gene number investigation in different treatments at different growth stages

Supplement: Supplemental Information 3 [file peerj-10-14488-s003.pdf]
