# Supplementary material for: Physiological changes and gene responses during Ganoderma lucidum growth with selenium supplementation
Source: PeerJ. 2022 Dec 20;10:e14488. doi: 10.7717/peerj.14488 (PMC9784338; doi:10.7717/peerj.14488)
Supplement: Supplemental Information 4 [file peerj-10-14488-s004.doc]

**Table S1 Throughput and quality of Illumina sequencing of *G. lucidum* transcriptome**

| Sample | Raw Reads | Clean Reads | Clean bases (bp) | N (%) | Q20 (%) | Q30 (%) | GC(%) |
| --- | --- | --- | --- | --- | --- | --- | --- |
| GCK2-1 | 29141952 | 28764004 | 4.14E+09 | 0.005903 | 97.53 | 93.66 | 57.30 |
| GCK2-2 | 30360566 | 30066700 | 4.31E+09 | 0.002182 | 97.65 | 93.91 | 59.15 |
| GCK2-3 | 33286858 | 32919194 | 4.71E+09 | 0.002157 | 97.53 | 93.74 | 57.15 |
| GCK4-1 | 32545574 | 32277856 | 4.71E+09 | 0.000571 | 98.43 | 95.99 | 60.21 |
| GCK4-2 | 30188248 | 29846282 | 4.32E+09 | 0.000565 | 98.53 | 96.21 | 58.11 |
| GCK4-3 | 27545438 | 27343434 | 4.03E+09 | 0.003587 | 98.60 | 96.24 | 60.14 |
| G2002-1 | 33103896 | 32785706 | 4.73E+09 | 0.005651 | 97.59 | 93.78 | 59.10 |
| G2002-2 | 33358116 | 32876766 | 4.71E+09 | 0.002224 | 97.60 | 93.86 | 56.49 |
| G2002-3 | 31141988 | 30805762 | 4.46E+09 | 0.002135 | 97.59 | 93.78 | 58.13 |
| G2004-1 | 33709980 | 33456314 | 4.88E+09 | 0.000965 | 98.47 | 96.10 | 60.33 |
| G2004-2 | 28439956 | 28233632 | 4.11E+09 | 0.003831 | 98.62 | 96.17 | 60.80 |
| G2004-3 | 29694980 | 29454604 | 4.28E+09 | 0.000931 | 98.53 | 96.12 | 61.20 |
