# Supplementary material for: Physiological changes and gene responses during Ganoderma lucidum growth with selenium supplementation
Source: PeerJ. 2022 Dec 20;10:e14488. doi: 10.7717/peerj.14488 (PMC9784338; doi:10.7717/peerj.14488)
Supplement: Supplemental Information 5 [file peerj-10-14488-s005.doc]

**Table S1 GO-terms with more than 10% genes in GO database of transcriptome**

| Catergory | GO_Term (Amount) |
| --- | --- |
| Biological process | GO:0008152 metabolic process (2760); GO:0009987 cellular process (2142);  GO:0071704 organic substance metabolic process (1905); GO:0044238 primary metabolic process (1789);  GO:0044699 single-organism process (1760); GO:0044237 cellular metabolic process (1398);  GO:0043170 macromolecule metabolic process (1221); GO:0044710 single-organism metabolic process (1140);  GO:0044763 single-organism cellular process (1098); GO:0044260 cellular macromolecule metabolic process (956);  GO:0019538 protein metabolic process (855); GO:0006807 nitrogen compound metabolic process (832);  GO:0034641 cellular nitrogen compound metabolic process (710); GO:0044267 cellular protein metabolic process (615);  GO:0009058 biosynthetic process (612); GO:0055114 oxidation-reduction process (608);  GO:0051179 localization (591); GO:0051234 establishment of localization 589);  GO:0006810 transport (587); GO:0046483 heterocycle metabolic process (558);  GO:1901576 organic substance biosynthetic process (558); GO:0006725 cellular aromatic compound metabolic process (553) |
| Cellular Component | GO:0005623 cell (1228); GO:0044464 cell part (1228);  GO:0005622 intracellular (1182); GO:0044424 intracellular part (916); GO:0043226 organelle (702); GO:0043229 intracellular organelle (702); GO:0016020 membrane (588) |
| Molecular  function | GO:0005488 binding (3281); GO:0003824 catalytic activity (3103);  GO:0043167 ion binding (1968); GO:0097159 organic cyclic compound binding (1953);  GO:1901363 heterocyclic compound binding (1952); GO:0016787 hydrolase activity (1089);  GO:0036094 small molecule binding (1056); GO:0043169 cation binding (1051);  GO:0000166 nucleotide binding (1037); GO:1901265 nucleoside phosphate binding (1037);  GO:0043168 anion binding (986); GO:0046872 metal ion binding (960);  GO:0003676 nucleic acid binding (876); GO:0046914 transition metal ion binding (859);  GO:0016491 oxidoreductase activity (838); GO:0097367 carbohydrate derivative binding (785);  GO:0032553 ribonucleotide binding (777); GO:0017076 purine nucleotide binding (755);  GO:0001882 nucleoside binding (745); GO:0032549 ribonucleoside binding (744);  GO:0001883 purine nucleoside binding (740); GO:0032550 purine ribonucleoside binding (740);  GO:0032555 purine ribonucleotide binding (740); GO:0035639 purine ribonucleoside triphosphate binding (739);  GO:0016740 transferase activity (727); GO:0030554 adenyl nucleotide binding (646);  GO:0032559 adenyl ribonucleotide binding (645); GO:0005524 ATP binding (644);  GO:0008270 zinc ion binding (598) |
