# Supplementary material for: Physiological changes and gene responses during Ganoderma lucidum growth with selenium supplementation
Source: PeerJ. 2022 Dec 20;10:e14488. doi: 10.7717/peerj.14488 (PMC9784338; doi:10.7717/peerj.14488)
Supplement: Supplemental Information 6 [file peerj-10-14488-s006.doc]

**Table S2 KEGG pathways with more than 10 up-regulated genes in each treatment**

| Pathway_ID | Pathway | GCKb Up Number | G200b Up Number | GCKm Up Number | G200m Up Number |
| --- | --- | --- | --- | --- | --- |
| ko01230 | Metabolism/Overview/Biosynthesis of amino acids | 17 | 18 | - | 12 |
| ko01200 | Metabolism/Overview/Carbon metabolism | 12 | 32 | 10 | - |
| ko00260 | Metabolism/Amino acid metabolism/Glycine, serine and threonine metabolism | 10 | - | - | - |
| ko00020 | Metabolism/Carbohydrate metabolism/Citrate cycle (TCA cycle) | - | 18 | - | - |
| ko00630 | Metabolism/Carbohydrate metabolism/Glyoxylate and dicarboxylate metabolism | - | 15 | - | - |
| ko00620 | Metabolism/Carbohydrate metabolism/Pyruvate metabolism | - | 15 | - | - |
| ko04146 | Cellular Processes/Transport and catabolism/Peroxisome | - | 15 | 13 | - |
| ko00500 | Metabolism/Carbohydrate metabolism/Starch and sucrose metabolism | - | 14 | - | - |
| ko04011 | Environmental Information Processing/Signal transduction /MAPK signaling pathway-yeast | - | 14 | - | - |
| ko00190 | Metabolism/Energy metabolism/Oxidative phosphorylation | - | 14 | - | - |
| ko03040 | Genetic Information Processing/Transcription/Spliceosome | - | 13 | - | - |
| ko00280 | Metabolism/Amino acid metabolism/Valine, leucine and isoleucine degradation | - | 13 | - | - |
| ko00010 | Metabolism/Carbohydrate metabolism/Glycolysis & Gluconeogenesis | - | 12 | - | - |
| ko00520 | Metabolism/Carbohydrate metabolism/Amino sugar and nucleotide sugar metabolism | - | 12 | 11 | - |
| ko00640 | Metabolism/Carbohydrate metabolism/Propanoate metabolism | - | 12 | - | - |
| ko01210 | Metabolism/Overview/2-Oxocarboxylic acid metabolism | - | 11 | - | - |
| ko04071 | Environmental Information Processing/Signal transduction/Sphingolipid signaling pathway | - | 11 | - | - |
| ko03013 | Genetic Information Processing/Translation/RNA transport | - | 10 | - | - |
| ko04212 | Organismal Systems /Aging /Longevity regulating pathway-worm | - | 10 | - | - |
| ko01212 | Metabolism/Overview/Fatty acid metabolism | - | 10 | - | - |
| ko00230 | Metabolism/Nucleotide metabolism/Purine metabolism | - | - | - | 10 |
| ko00240 | Metabolism/Nucleotide metabolism/Pyrimidine metabolism | - | - | - | 10 |
