# Supplementary material for: Physiological changes and gene responses during Ganoderma lucidum growth with selenium supplementation
Source: PeerJ. 2022 Dec 20;10:e14488. doi: 10.7717/peerj.14488 (PMC9784338; doi:10.7717/peerj.14488)
Supplement: Supplemental Information 7 — The genes of the left part are the top 10 up-regulated genes treated with selenium compared to the control group at budding and mature stages. While the right-part-genes are the top 10 down-regulated genes. [file peerj-10-14488-s007.doc]

**Table S3 The top 10 genes with largest difference treated with selenium**

| Gene ID | GCK b | G200 b | pvalue | GO | KEGG | Gene ID | GCK b | G200 b | pvalue | GO | KEGG |
| --- | --- | --- | --- | --- | --- | --- | --- | --- | --- | --- | --- |
| GL23959-G | 0.96 | 615.88 | 3.19E-34 | GO:0004806, etc. | - | GL15942-G | 1169.30 | 10.68 | 9.69E-28 | GO:0009277, etc. | - |
| GL25320-G | 114.05 | 10589.91 | 1.99E-30 | GO:0016491, etc. | - | GL23380-G | 3895.42 | 52.02 | 1.53E-27 | - | - |
| **GL23263-G** | **278.75** | **39807.93** | **1.15E-25** | **GO:0003824, etc.** | **-** | GL22366-G | 1503.90 | 22.74 | 2.44E-25 | - | - |
| GL19980-G | 20.99 | 1067.21 | 3.83E-24 | - | - | GL15952-G | 1790.18 | 54.13 | 1.67E-19 | - | - |
| GL29157-G | 2.55 | 315.87 | 1.02E-23 | - | - | GL21310-G | 934.65 | 32.59 | 5.10E-18 | - | - |
| GL26673-G | 7.93 | 452.79 | 4.22E-22 | - | - | GL18877-G | 509.36 | 17.01 | 2.57E-17 | GO:0009277, etc. | - |
| GL23603-G | 70.05 | 2374.00 | 8.84E-21 | - | - | GL16428-G | 1141.64 | 56.25 | 2.25E-16 | GO:0004185, etc. | - |
| GL15998-G | 32.92 | 1062.46 | 4.56E-20 | - | - | GL16427-G | 1936.66 | 108.87 | 7.74E-16 | GO:0004185, etc. | - |
| GL20423-G | 4.28 | 245.79 | 8.25E-20 | - | - | GL27487-G | 863.52 | 31.19 | 1.40E-15 | - | - |
| GL25269-G | 3.06 | 3073.04 | 2.28E-19 | GO:0006950 | - | GL28719-G | 1053.26 | 59.50 | 5.54E-15 | - | - |
| Gene ID | GCK m | G200 m | pvalue | GO | KEGG | Gene ID | GCK m | G200 m | pvalue | GO | KEGG |
| GL26604-G | 12.13 | 593.17 | 2.29E-17 | GO:0005740, etc. | K00419 | GL23959-G | 586.89 | 3.14 | 9.22E-24 | GO:0004806, etc. | - |
| GL30187-G | 7.16 | 337.65 | 1.34E-12 | - | K11097 | GL24094-G | 221.11 | 0.59 | 5.54E-21 | - | - |
| GL29413-G | 56.64 | 1188.29 | 5.98E-11 | GO:0016020, etc. | K07342 | GL17265-G | 4774.05 | 82.16 | 5.71E-19 | - | - |
| GL18309-G | 735.01 | 11241.07 | 6.10E-10 | GO:0004609, etc. | - | **GL24771-G** | **30108.93** | **526.63** | **9.22E-19** | **GO:0003824, etc.** | **-** |
| GL16582-G | 9.83 | 122.90 | 6.85E-09 | - | - | GL28244-G | 224.49 | 3.27 | 3.30E-16 | - | - |
| GL24883-G | 572.05 | 5600.35 | 1.14E-08 | GO:0004497, etc. | - | GL27710-G | 185.85 | 2.74 | 3.33E-16 | - | - |
| GL21103-G | 3.46 | 62.89 | 3.70E-08 | - | - | GL24927-G | 13287.08 | 454.48 | 2.52E-15 | GO:0000166, etc. | K08900 |
| GL31354-G | 4.93 | 60.24 | 4.96E-07 | - | - | GL24498-G | 199.80 | 0.00 | 2.77E-15 | GO:0016757 | - |
| GL29437-G | 58.39 | 464.38 | 5.25E-07 | - | K12624 | GL29866-G | 444.35 | 7.92 | 3.07E-15 | - | - |
| GL24211-G | 31.14 | 293.97 | 9.06E-07 | - | - | GL30823-G | 310.07 | 8.34 | 7.21E-15 | GO:0016998 | - |

The genes of the left part are the top 10 up-regulated genes treated with selenium compared to the control group at budding and mature stages. While the right-part-genes are the top 10 down-regulated genes.
