# Supplementary material for: Physiological changes and gene responses during Ganoderma lucidum growth with selenium supplementation
Source: PeerJ. 2022 Dec 20;10:e14488. doi: 10.7717/peerj.14488 (PMC9784338; doi:10.7717/peerj.14488)
Supplement: Supplemental Information 8 — The genes of the left part are the top 10 genes with the highest expression treated with selenium compared to the control group at budding and mature stages. While the right-part-genes are the control group’s. [file peerj-10-14488-s008.doc]

**Table S5 The top 10 genes with highest expression in different treatments**

| Gene ID | GCK b | G200 b | pvalue | GO | KEGG | Gene ID | GCK b | G200 b | pvalue | GO | KEGG |
| --- | --- | --- | --- | --- | --- | --- | --- | --- | --- | --- | --- |
| GL21838-G | 4449.64 | 89546.7 | 9.77E-06 | GO:0004497, etc. | - | GL22189-G | 61301.00 | 28873.79 | 4.16E-02 | GO:0004096, etc. | K03781 |
| GL19520-G | 3048.06 | 43709.8 | 1.43E-03 | - | - | GL22047-G | 50549.00 | 8104.43 | 3.99E-07 | - | - |
| **GL23263-G** | **278.75** | **39807.9** | **1.15E-25** | **GO:0003824, etc.** | **-** | GL23307-G | 42344.00 | 634.56 | 4.39E-09 | - | - |
| GL30277-G | 6727.78 | 33577.8 | 1.53E-02 | - | - | GL23779-G | 36623.00 | 14819.54 | 5.69E-03 | - | - |
| GL31403-G | 968.58 | 32952.7 | 1.42E-07 | GO:0004497, etc. | - | GL21313-G | 34040.00 | 16612.37 | 3.11E-02 | GO:0003824, etc. | K00134 |
| GL25942-G | 341.96 | 29124.9 | 3.21E-03 | - | - | GL25927-G | 29986.00 | 425.55 | 7.15E-13 | - | - |
| GL31371-G | 4417.27 | 25655.6 | 4.18E-05 | - | - | GL26621-G | 23802.00 | 8920.29 | 2.03E-03 | - | - |
| GL28560-G | 4020.52 | 25504.7 | 6.32E-05 | - | - | GL24338-G | 22428.00 | 1849.30 | 4.41E-06 | - | - |
| GL29481-G | 3558.39 | 25476.7 | 3.36E-04 | - | - | GL30114-G | 21981.00 | 5662.02 | 4.52E-05 | GO:0000015, etc. | K01689 |
| GL21985-G | 7652.01 | 23178.5 | 2.31E-02 | GO:0005622, etc. | K04513 | GL20810-G | 15295.00 | 5247.15 | 1.92E-03 | GO:0009058 | - |
| Gene ID | GCK m | G200 m | pvalue | GO | KEGG | Gene ID | GCK m | G200 m | pvalue | GO | KEGG |
| GL23307-G | 2419.26 | 100685.00 | 2.13E-03 | - | - | GL25963-G | 74626.80 | 21166.70 | 9.39E-04 | GO:0016020, etc. | K08176 |
| GL20732-G | 1600.72 | 97567.60 | 4.80E-06 | - | - | GL30277-G | 55006.70 | 7243.66 | 5.27E-06 | - | - |
| GL22047-G | 8075.91 | 53744.60 | 1.58E-03 | - | - | GL21985-G | 44021.00 | 7814.57 | 9.11E-06 | GO:0005622, etc. | K04513 |
| GL30174-G | 14330.08 | 40116.40 | 1.54E-02 | GO:0016491, etc. | K00128 | GL22875-G | 43993.40 | 17381.30 | 1.39E-02 | GO:0000785, etc. | K03235 |
| GL30114-G | 7702.55 | 25809.30 | 1.30E-03 | GO:0000015, etc. | K01689 | GL20764-G | 37981.60 | 1666.80 | 3.72E-05 | GO:0003824, etc. | K13953 |
| GL28232-G | 3090.59 | 20558.40 | 9.23E-05 | - | - | GL31371-G | 37391.40 | 3572.35 | 6.78E-09 | - | - |
| GL22160-G | 6233.45 | 19442.90 | 2.55E-02 | - | - | GL21004-G | 37303.50 | 973.03 | 2.47E-09 | - | - |
| GL17001-G | 6261.10 | 14487.60 | 1.83E-02 | GO:0003824, etc. | K17818 | GL28560-G | 37180.70 | 5662.93 | 1.38E-06 | - | - |
| GL22109-G | 5192.59 | 13983.20 | 7.84E-03 | - | - | GL24688-G | 35785.90 | 7505.42 | 2.91E-05 | GO:0005524 | K03283 |
| GL24338-G | 4061.54 | 12522.00 | 1.96E-03 | - | - | **GL24771-G** | **30108.90** | **526.63** | **9.22E-19** | **GO:0003824, etc.** | **-** |

The genes of the left part are the top 10 genes with the highest expression treated with selenium compared to the control group at budding and mature stages. While the right-part-genes are the control group’s.
